# Neuronal exosomal miR-25-3p attenuates M1 microglial activation and neurotoxicity by targeting TLR4 to regulate the NF-κB signaling pathway

**DOI:** 10.3389/fneur.2026.1803653

**Published:** 2026-06-03

**Authors:** Guangjun Hu, Sarulatuya, Siyu Du, Zhile Huang, Xinyi Tang

**Affiliations:** Department of Anesthesiology, Wuhan Third Hospital/Tongren Hospital of Wuhan University, Wuhan, Hubei Province, China

**Keywords:** exosomes, microglial polarization, MiR-25-3p, neuroinflammation, perioperative neurocognitive disorders, TLR4/NF-κB

## Abstract

**Background:**

Perioperative neurocognitive disorders (PND) are severe postoperative complications in the elderly, with neuroinflammation driven by pro-inflammatory M1 microglia being a core pathological mechanism. This study aimed to investigate the role of neuron-derived exosomes in regulating microglial polarization and its underlying molecular mechanism.

**Methods:**

We utilized a neuron–microglia transwell co-culture system. By modulating microRNA-25-3p (miR-25-3p) in neurons or Toll-like receptor 4 (TLR4) in microglia, we assessed microglial M1/M2 polarization, TLR4/NF-κB pathway activation, and subsequent neurotoxicity using qRT-PCR, Western Blot, flow cytometry, and dual-luciferase reporter assays.

**Results:**

Neuronal exosomes were effectively internalized by microglia. Exosomal miR-25-3p suppressed the activation of the TLR4/MyD88/NF-κB signaling pathway by directly targeting the 3’-UTR of TLR4. This significantly inhibited microglial polarization towards the pro-inflammatory M1 phenotype and reduced the release of pro-inflammatory cytokines. Functionally, this process attenuated M1 microglia-mediated neuronal apoptosis, oxidative stress, and functional impairment. Direct manipulation of TLR4 expression in microglia confirmed its pivotal role in this regulatory axis.

**Conclusion:**

Our findings systematically demonstrate that neurons can release exosomes carrying miR-25-3p to target and suppress the microglial TLR4/NF-κB signaling pathway, thereby inhibiting M1 polarization and alleviating neurotoxicity. This discovery deepens the understanding of PND pathophysiology and provides a theoretical basis and potential therapeutic targets for novel PND treatment strategies targeting neuron–glia communication.

## 1 Introduction

With the accelerating trend of global population aging and the popularization of surgical procedures, perioperative neurocognitive disorders (PND) have become one of the most common postoperative complications in elderly patients (1, 2). The clinical manifestations of PND are diverse, ranging from short-term postoperative delirium to long-term cognitive decline, which seriously affect the postoperative patients’ postoperative recovery and quality of life, while also being linked to a higher mortality risk, bringing a significant socioeconomic and healthcare burden (3). Although the pathogenesis of PND remains incompletely understood, accumulating evidence indicates that neuroinflammation is a critical factor in its development.

Neuroinflammation is considered to be the core pathophysiological mechanism of the occurrence and development of PND (1, 4). Microglia, the primary immune cells residing in the central nervous system (CNS), exert a “double-edged sword” function in both initiating and modulating neuroinflammation (5). Under the stimulation of different pathological microenvironments, microglia can polarize into two distinct functional phenotypes: the classical M1 phenotype (pro-inflammatory and neurotoxic) and the alternative M2 phenotype (anti-inflammatory and neuroprotective) (6). In the course of PND, over-activated M1 microglia exacerbate neuronal damage, impair synaptic plasticity, which culminates in cognitive impairment by releasing a large number of a variety of pro-inflammatory cytokines, including tumor necrosis factor-*α* (TNF-α), interleukin-1β (IL-1β), and interleukin-6 (IL-6) (6, 7). Therefore, inhibiting the excessive polarization of microglia to the M1 type and promoting their transformation to the M2 protective phenotype has become an important therapeutic approach for preventing and managing PND (6).

Among the many signaling pathways that drive M1 polarization of microglia, the signaling pathway mediated by Toll-like receptor 4 (TLR4) has a pivotal function (8). As a pattern recognition receptor, TLR4 is highly expressed on the microglial cell surface, where it identifies damage-associated molecular patterns (DAMPs), thereby activating the downstream nuclear factor-kappa B (NF-κB) signaling cascade (1, 9). After entering the nucleus, NF-κB triggers the transcription of multiple pro-inflammatory genes, thereby driving the M1 activation of microglia and amplifying the inflammatory response in a cascade (10, 11). It has been confirmed that the TLR4/NF-κB signaling pathway is significantly activated in a model of cognitive dysfunction induced by sevoflurane (8, 10). Therefore, targeted regulation of the TLR4/NF-κB pathway may provide an effective therapeutic target for intervening in PND.

In recent years, extracellular vesicles (EVs), especially exosomes, as emerging facilitators of intercellular signaling, have played a significant function in both the normal and disease-related processes within the CNS (12). As cell-secreted nanoscale vesicles, exosomes typically have a diameter ranging from 30 to 150 nm, and are encapsulated with a cargo of bioactive substances, including proteins, lipids, and various nucleic acids like microRNAs (miRNAs). These vesicles possess the ability to traverse the blood–brain barrier, enabling them to deliver these molecules to target cells, thereby regulating their functions (13). Neurons can secrete exosomes and communicate with neighboring microglia by transporting specific miRNAs, thereby affecting the process of neuroinflammation (13, 14). This neuron–glia communication provides a novel viewpoint for comprehending the maintenance of CNS homeostasis and the occurrence of diseases.

MicroRNA-25-3p (miR-25-3p) is a non-coding single-stranded RNA which has a crucial regulatory function in numerous diseases. Studies have shown that miR-25-3p plays a significant function in several pathological conditions, including the inflammatory response, cellular apoptosis, and oxidative stress (15). It has been found that miR-25-3p can directly target TLR4 and negatively modulate its expression levels, thereby exerting anti-inflammatory and neuroprotective effects in sepsis-associated encephalopathy (16). Our previous dual-luciferase assay confirmed the direct interaction of miR-25-3p with TLR4 and negatively regulate its expression (Figure S1).

Based on this, we hypothesized that neurons may release miR-25-3p through exosomes, which is taken up by microglia. After this uptake, miR-25-3p is able to directly target and suppress the production of TLR4, consequently impeding the activation of the NF-κB signaling cascade, and finally suppressing the differentiation of microglia to the M1 type, reducing neuroinflammation and neuronal damage. The present investigation was undertaken to systematically elucidate the functional role and underlying mechanism of the “neuronal exosomal miR-25-3p/TLR4/NF-κB” signaling axis in regulating microglial polarization and neurotoxicity, in order to provide a new theoretical basis and potential therapeutic targets for the prevention and treatment of PND.

## 2 Materials and methods

### 2.1 Cell culture

The murine hippocampal neuronal cell line HT22 and the BV2 microglial cell line were procured from the Cell Bank of the Chinese Academy of Sciences (Shanghai, China). HT22 cells were maintained in high-glucose Dulbecco’s Modified Eagle Medium (DMEM; Gibco) containing with 10% fetal bovine serum (FBS; Gibco, USA), as well as a combination of 100 U/mL penicillin, and 100 μg/mL streptomycin (Gibco). BV2 cells were grown in DMEM/F12 medium (Gibco) that was supplemented with 10% exosome-depleted FBS (Exo-FBS™; System Biosciences, Palo Alto, CA, USA), and an antibiotic mixture of 100 U/mL penicillin, and 100 μg/mL streptomycin. All cells were maintained in a cell incubator at 37 °C with 5% CO₂.

### 2.2 Exosome isolation, identification, and labeling

Exosome Isolation and Purification: When HT22 cells achieved approximately 80% confluence, the growth medium was exchanged for fresh medium containing 10% Exo-FBS™ and incubation continued for an additional 24 h. To eliminate cells and cellular debris, the collected supernatant underwent sequential centrifugation, first at 300 × g for 10 min and then at 12,000 × g for 30 min at 4 °C. Following this, the resulting supernatant was passed through a 0.22 μm filter (Millipore, Burlington, MA, USA) and then subjected to ultracentrifugation at 100,000 × g for 70 min at 4 °C utilizing an ultracentrifuge (Beckman Coulter, Brea, CA, USA). The resulting pellet was resuspended in PBS and put through a final ultracentrifugation step at 100,000 × g for 70 min to obtain the final exosome pellet (Sev-Exos). The total protein content of the isolated exosomes was determined with a BCA protein assay kit (Beyotime, Shanghai, China).

Exosome Identification: The size distribution profile of the exosomes was determined by a ZetaView® Nanoparticle Tracking Analyzer (NTA, Particle Metrix, Inning am Ammersee, Germany). The presence of exosome markers CD9, CD81, and TSG101 was confirmed via Western blot.

Exosome Fluorescent Labeling and Uptake Assay: Exosomes were tagged using the PKH67 green fluorescent dye (Sigma-Aldrich, St. Louis, MO, USA). To eliminate unbound dye, the resulting labeled exosomes were ultracentrifuged at 100,000 × g to remove excess dye, resuspended, and then co-incubated with BV2 cells. The uptake of Sev-Exos was visualized using a fluorescence microscope (Leica, Wetzlar, Germany).

Exosome Secretion Inhibition: To verify the role of exosomes in mediating neuron–microglia communication, the neutral sphingomyelinase inhibitor GW4869 (20 μM, Sigma-Aldrich) was utilized to block exosome release from HT22 cells. HT22 cells were pre-treated with GW4869 for 12 h prior to co-culture or exosome extraction, as previously described (17).

### 2.3 Transwell co-culture and cell intervention

A transwell polycarbonate membrane cell inserted with a pore size of 0.4 μm (Corning Costar, Corning, NY, USA) was employed for the co-culture experiments. For the setup, HT22 cells were plated in the bottom chamber of a 24-well plate at a density of 1 × 10^5^ cells/well, and BV2 cells were added to the top chamber at a density of 5 × 10^4^ cells/well. According to the experimental design, the designated cells were transfected:

To investigate the function of miR-25-3p, miR-25-3p mimic, mimic-NC, miR-25-3p inhibitor, or inhibitor-NC (RiboBio, Guangzhou, China) was introduced into the HT22 cells located in the bottom chamber with Lipofectamine 3,000 (Invitrogen, Carlsbad, CA, USA). To examine the function of TLR4, siRNA-TLR4, scr siRNA, pcDNA3.1-TLR4, or the empty vector pcDNA3.1 (GenePharma, Shanghai, China) was delivered into the BV2 cells situated in the top chamber. After 6 h of transfection, the culture medium was replaced, and the co-culture was maintained for 48 h prior to harvesting the cells or their supernatants for subsequent analysis.

### 2.4 Cell viability assay

The viability of the cells was evaluated using a CCK-8 kit (Dojindo, Kumamoto, Japan). Cells were plated in a 96-well plate at a concentration of 5 × 10^3^ cells/well. Following the indicated treatment, 10 μL of CCK-8 solution was dispensed into each well, and the plates were incubated for an additional 2 h. The absorbance was subsequently read at a wavelength of 450 nm with a microplate reader (BioTek, Winooski, VT, USA).

### 2.5 Quantitative real-time polymerase chain reaction (qRT-PCR)

Isolation of total RNA from cells was accomplished with TRIzol reagent (Invitrogen). A NanoDrop 2000 spectrophotometer (Thermo Fisher Scientific, Waltham, MA, USA) was utilized to assess the concentration and purity of the isolated RNA. For mRNA detection, cDNA was synthesized from 1 μg of total RNA via reverse transcription with the PrimeScript™ RT Master Mix (Takara, Shiga, Japan). For miRNA detection, the Mir-X™ miRNA First-Strand Synthesis Kit (Takara) was employed for the reverse transcription step. QRT-PCR analysis was conducted with TB Green® Premix Ex Taq™ II (Takara) on a CFX96 Touch Real-Time PCR Detection System (Bio-Rad, Hercules, CA, USA). The reaction program was: 95 °C for 30 s, followed by 40 cycles of 95 °C for 5 s and 60 °C for 30 s. The relative expression was calculated using the 2^-ΔΔCt^ method, with GAPDH as the internal control for mRNA and U6 for miRNA. All primers were procured from Sangon Biotech (Shanghai) Co., Ltd., with their specific sequences listed in Table 1.

**Table 1:** Primer sequences used in the qRT-PCR experiments.

Gene	Forward primer (5′-3′)	Reverse primer (5′-3′)
*TLR4*	TTTATTCAGAGCCGTTGGTG	CAGAGGATTGTCCTCCCATT
MyD88	ATCGCTGTTCTTGAACCCTCG	CTCACGGTCTAACAAGGCCAG
*NF-κB (p65)*	CTTCCTCAGCCATGGTACCTCT	CAAGTCTTCATCAGCATCAAACTG
iNOS	ACATCGACCCGTCCACAGTAT	CAGAGGGGTAGGCTTGTCTC
MCP-1	GCTACAAGAGGATCACCAGCAG	GTCTGGACCCATTCCTTCTTGG
*Arg1*	CTCCAAGCCAAAGTCCTTAGAG	GGAGCTGTCATTAGGGACATCA
*Fizz-1*	CAAGGAACTTCTTGCCAATCCAG	CCAAGATCCACAGGCAAAGCCA
TRAF6	TTTCCCTGACGGTAAAGTGCCC	ACCTGGCACTTCTGGAAAGGAC
Caspase3	GGAGTCTGACTGGAAAGCCGAA	CTTCTGGCAAGCCATCTCCTCA
BCL-2	CTCCAAGCCAAAGTCCTTAGAG	GGAGCTGTCATTAGGGACATCA
BCL-XL	GGTTGCCAAGCCTTATCGGAAATG	GCCGCATCCTGAGGGTCTTC
NeuN	TACGCAGCCTACAGATACGCTC	TGGTTCCAATGCTGTAGGTCGC
MAP2	AGGCTGTAGCAGTCCTGAAAGG	CTTCCTCCACTGTGACAGTCTG
BDNF	CATCCGAGGACAAGGTGGCTTG	GCCGAACTTCTGGTCGTCTATC
Synapsin I	CGATGCCAAATATGACGTGCGTG	AGCATGCGAGCCCAGTATTGG
PSD-95	TCCACTCTGACAGTGAGACCGA	CGTCACTGTCTCGTAGCTCAGA
*miR-25-3p*	CCGCATTGCACTTGTCTCG	GTCGTATCCAGTGCAGGGTCCGAGG TATTCGCACTGGATACGAC TCAGAC
GAPDH	CAACGTGTCAGTGGTGGACCTG	GTGTCGCTGTTGAAGTCAGAGGAG
*U6*	CTCGCTTCGGCAGCACATATACT	ACGCTTCACGAATTTGCGTGTC

### 2.6 Western blot analysis

Cellular proteins were isolated using RIPA lysis buffer (Beyotime), and the protein levels were quantified with the BCA method. Equivalent quantities of protein for each sample were loaded, resolved on a 10% SDS-PAGE gel, and then electroblotted onto a PVDF membrane (Millipore). Following the transfer, the membranes were blocked for 1 h at room temperature with a solution of 5% non-fat milk, followed by an overnight incubation at 4 °C with the appropriate primary antibody. Subsequent to washing with TBST, the membranes were treated with a horseradish peroxidase (HRP)-labeled secondary antibody for 1 h at ambient temperature. Detection of the protein bands was achieved with an ECL chemiluminescence kit (Beyotime), and densitometric analysis of the bands was conducted using ImageJ software. Comprehensive details regarding all antibodies used are provided in Table 2.

**Table 2:** Information on Western blot antibodies.

Target protein	Article number	Dilution rate	Supplier
TLR4	14,358	1:1000	Cell Signaling Technology (Danvers, MA, USA)
MyD88	3,699	1:1000	Cell Signaling Technology (Danvers, MA, USA)
NF-κB p65	8,242	1:1000	Cell Signaling Technology (Danvers, MA, USA)
TRAF6	TA329145	1:1000–1:2000	OriGene Technologies (Rockville, MD, USA)
iNOS	ab178945	1:1000	Abcam (Cambridge, MA, USA)
Arg1	ab91279	1:1000	Abcam (Cambridge, MA, USA)
MCP-1	ab9669	1:500–1:2000	Abcam (Cambridge, UK)
Fizz-1	ab128859	1:1000–1:5000	Abcam (Cambridge, UK)
CD81	ab109201	1:1000	Abcam (Cambridge, MA, USA)
CD9	ab92726	1:1000	Abcam (Cambridge, MA, USA)
TSG101	ab125011	1:1000	Abcam (Cambridge, MA, USA)
Caspase3	9,662	1:1000	Cell Signaling Technology (Danvers, MA, USA)
BCL-2	2,876	1:1000	Cell Signaling Technology (Danvers, MA, USA)
BCL-XL	10,783-1-AP	1:1000	Proteintech (Chicago, IL, USA)
NeuN	ab177487	1:1000–1:5000	Abcam (Cambridge, UK)
MAP2	ab32454	1:500–1:2000	Abcam (Cambridge, UK)
BDNF	ab108319	1:1000–1:5000	Abcam (Cambridge, UK)
Synapsin I	ab64581	1:1000–1:5000	Abcam (Cambridge, UK)
PSD-95	ab18258	1:500–1:2000	Abcam (Cambridge, UK)
HO-1	43,966	1:1000	Cell Signaling Technology (Danvers, MA, USA)
NQO1	62,262	1:1000	Cell Signaling Technology (Danvers, MA, USA)
GCLC	48,005	1:1000	Cell Signaling Technology (Danvers, MA, USA)
Prx1	8,732	1:1000	Cell Signaling Technology (Danvers, MA, USA)
GAPDH	ab9485	1:2500	Abcam (Cambridge, UK)

### 2.7 Flow cytometry

For microglial phenotype analysis, BV2 cells were collected and prepared as a single-cell suspension in flow cytometry buffer (PBS containing 2% FBS). After incubating with an Fc receptor blocker (Anti-CD16/32; BioLegend, San Diego, CA, USA) for 10 min, FITC-CD86 (BioLegend), PE-CD206 (BioLegend), and APC-CD16/32 (BioLegend) antibodies were introduced, and the cells were subsequently incubated in darkness for 30 min at 4 °C.

To detect apoptosis, HT22 cells were stained using an Annexin V-FITC/PI Apoptosis Detection Kit (Dojindo). Data acquisition for all samples was carried out on a CytoFLEX flow cytometer (Beckman Coulter, Brea, CA, USA), with subsequent analysis performed via CytExpert software.

### 2.8 Enzyme-linked immunosorbent assay (ELISA)

To prepare samples for analysis, cell culture supernatants were harvested and subjected to centrifugation to eliminate cell debris. The levels of IL-6, TNF-*α*, IL-1*β*, IL-10, and TGF-β were quantified with ELISA kits (Dakewe, Shenzhen, China). The amounts of superoxide dismutase (SOD), malondialdehyde (MDA), and glutathione (GSH) in cell lysates were measured with ELISA kits (Jiancheng Bioengineering, Nanjing, China). All operations were performed strictly according to the manufacturer’s instructions. All kit information is detailed in Table 3.

**Table 3:** Information of ELISA kits.

Target protein	Article number	Supplier
Mouse TNF-α quantikine ELISA Kit	MTA00B	R&D Systems (Minneapolis, MN, USA)
Mouse IL-1β quantikine ELISA Kit	MLB00C	R&D Systems (Minneapolis, MN, USA)
Mouse IL-6 quantikine ELISA Kit	M6000B	R&D Systems (Minneapolis, MN, USA)
Mouse IL-10 ELISA Kit	ab100697	Abcam (Cambridge, MA, USA)
Mouse TGF-beta 1 ELISA Kit	ab119558	Abcam (Cambridge, UK)
Lipid peroxidation MDA assay kit	S0131S	Beyotime Biotechnology (Shanghai, China)
Superoxide dismutase activity assay kit	CS0009	Sigma-Aldrich (St. Louis, MO, USA)
Glutathione (GSH) ELISA Kit	ab206402	Abcam (Cambridge, UK)

### 2.9 Intracellular calcium measurement

Intracellular calcium levels in BV2 microglial cells were quantified using Fluo-4 a.m. fluorescent probe combined with flow cytometry. Logarithmic-phase BV2 cells were harvested and washed with pre-warmed calcium-free Hanks’ balanced salt solution (HBSS, pH 7.4). Cells were then resuspended at a density of 1 × 10^6^ cells/mL in HBSS containing 5 μM Fluo-4 a.m. (Thermo Fisher Scientific) and 0.02% Pluronic F-127, followed by incubation at 37 °C in the dark for 30 min. After incubation, cells were washed twice with HBSS to remove residual dye and resuspended in an appropriate volume of HBSS for analysis. Flow cytometric analysis was performed using a BD FACSCanto II flow cytometer with excitation at 488 nm, and fluorescence emission was collected through the FITC channel (530/30 nm). A minimum of 10,000 events were acquired per sample, and mean fluorescence intensity (MFI) was analyzed using FlowJo software.

### 2.10 Immunofluorescence staining

Cells were cultured in a confocal dish, received the indicated treatment, and were subsequently preserved with 4% paraformaldehyde for 15 min, The cells were then made permeable using 0.3% Triton X-100 for a duration of 15 min, followed by blocking for 1 h with a 5% BSA solution (TLR4, 1:200, Cat# 14358, Cell Signaling Technology; NF-κB p65, 1:200, Cat# 8242, Cell Signaling Technology; MyD88, 1:200, Cat# 4283, Cell Signaling Technology; TRAF6, 1:200, Cat# 8028, Cell Signaling Technology) were introduced, and the samples were kept at 4 °C through the night. Following a PBS wash, the cells were treated with Alexa Fluor 488 or 594-conjugated secondary antibodies (1:500; Invitrogen) and maintained at ambient temperature for a duration of one hour. DAPI (Beyotime) was utilized for nuclear counterstaining, and a laser scanning confocal microscope was employed to view and record the resulting images.

### 2.11 Dual-luciferase reporter assay

To confirm the direct interaction between miR-25-3p and the TLR4 3’-UTR, we generated a psiCHECK-2 dual-luciferase reporter plasmid (Promega, Madison, WI, USA) that contained either the wild-type (WT) or a mutated (MUT) version of the TLR4 3’-UTR sequence. HEK293T cells were placed into a 24-well plate for cultivation. Upon reaching 70% confluence, the cells were co-transfected with the reporter plasmid along with either miR-25-3p mimics or a negative control (NC) mimic using Lipofectamine 3,000. Forty-eight hours post-transfection, the activity of luciferase was quantified the Dual-Luciferase® Reporter Assay System (Promega, Madison, WI, USA). Renilla luciferase activity was used as an internal control to calculate the relative luciferase activity.

### 2.12 Statistical analysis

All experiments were independently repeated at least three times. Data are presented as the mean ± standard deviation (SD). Statistical evaluations were conducted utilizing GraphPad Prism version 9.0 (GraphPad Software, San Diego, CA, USA). An independent sample t-test was applied for making comparisons between two groups, while a one-way analysis of variance (ANOVA) succeeded by Tukey’s *post hoc* test was employed for evaluating multiple group comparisons. A result was deemed statistically significant when the *p*-value was less than 0.05.

## 3 Results

### 3.1 Identification of neuron-derived exosomes and their successful uptake by microglia

**Figure 1:**
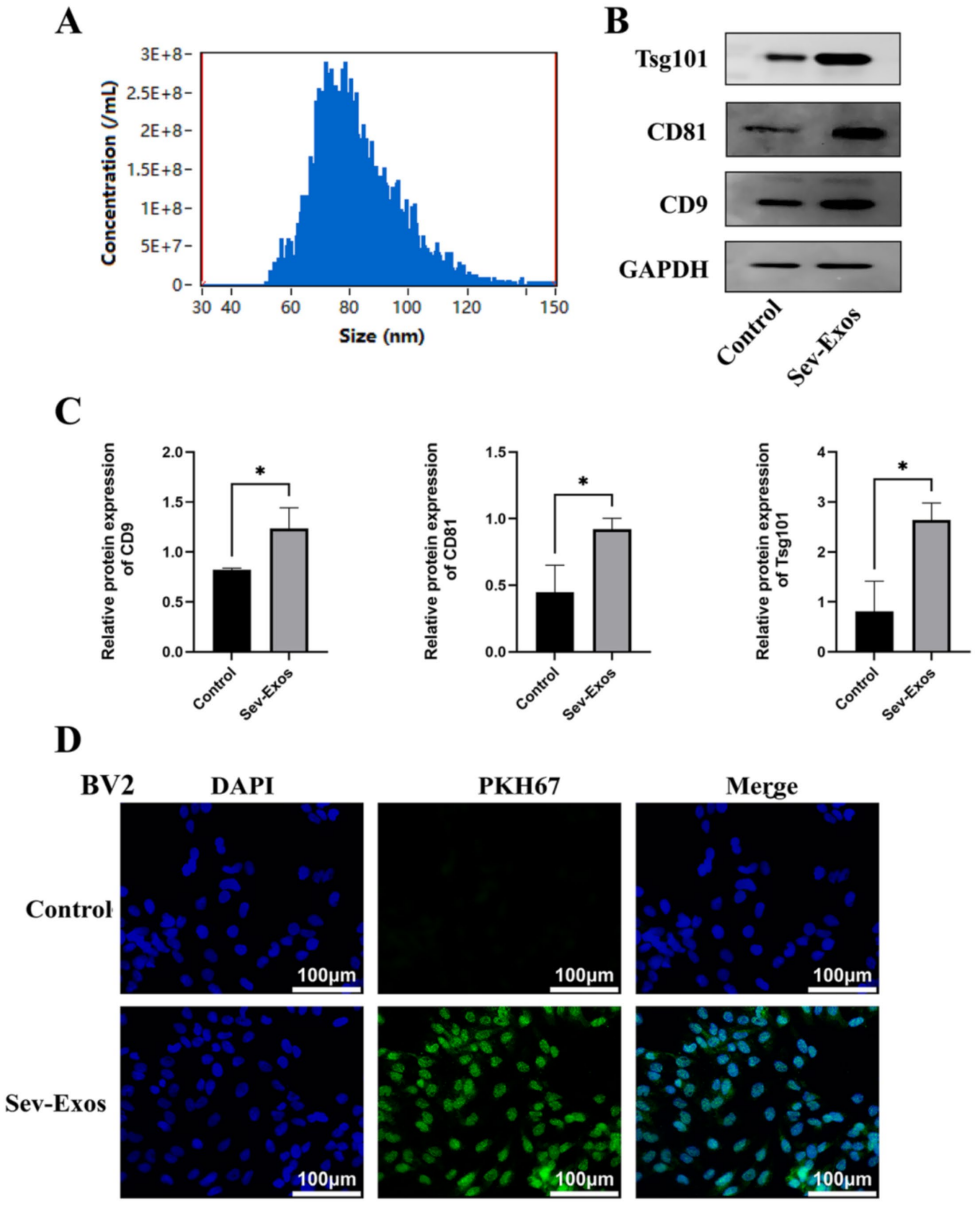
Characterization of neuron-derived exosomes (Sev-Exos) from HT22 cells and their uptake by BV2 microglia. **(A)** NTA showing the particle size distribution and concentration of the isolated exosomes. **(B)** Western blot analysis of exosome marker proteins (Tsg101, CD81, CD9) in cell lysates (Control) and purified exosomes (Sev-Exos). GAPDH served as a loading control. **(C)** Quantitative analysis of the relative protein expression of Tsg101, CD81, and CD9. **(D)** Representative fluorescence micrographs showing BV2 cells following co-incubation with PKH67-labeled exosomes (green). Nuclei were counterstained with DAPI (blue). Scale bar = 100 μm. Data are presented as mean ± SD (*n* = 3). *p < 0.05.

To investigate the communication between neurons and microglia, we first isolated Sev-Exos from the conditioned medium of HT22 hippocampal neuronal cells. NTA results showed that the isolated Sev-Exos had a particle size distribution mainly between 60–120 nm, with a peak particle size of about 80 nm, a finding that aligns with the characteristic size profile of exosomes (Figure 1A). Western blot analysis further confirmed that Sev-Exos highly expressed the exosomal marker proteins Tsg101, CD81, and CD9, indicating high purity of the exosomes (Figures 1B,C). To verify whether microglia can take up Sev-Exos, we co-cultured PKH67 fluorescent dye-labeled Sev-Exos with BV2 microglial cells. Fluorescence microscopy observation showed obvious green fluorescence signals in the cytoplasm of BV2 cells, indicating that neuron-derived exosomes can be effectively internalized by microglia (Figure 1D).

### 3.2 Neuronal Exosomal miR-25-3p inhibits M1 polarization of microglia

**Figure 2:**
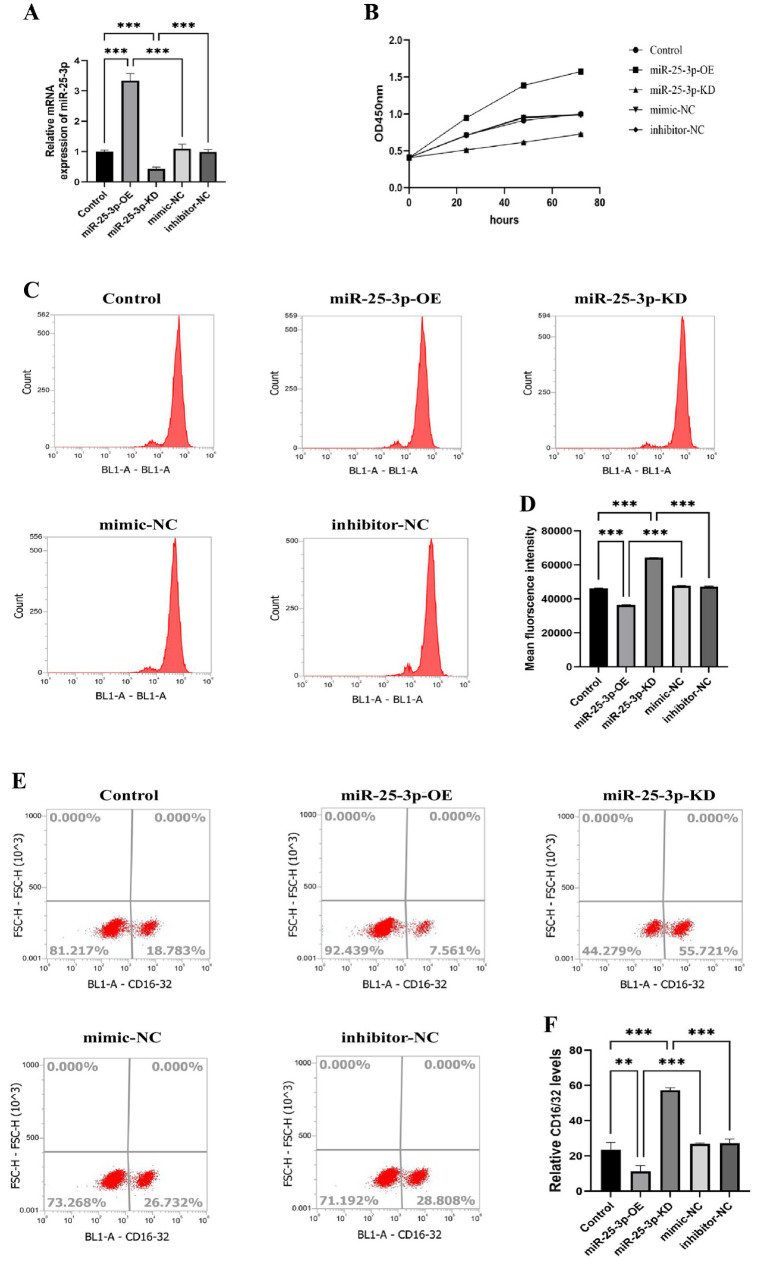
Neuronal exosomal miR-25-3p modulates the activation of BV2 microglia. BV2 cells were co-cultured with HT22 neurons transfected with miR-25-3p mimic (miR-25-3p-OE), miR-25-3p inhibitor (miR-25-3p-KD), or their corresponding negative controls (mimic-NC, inhibitor-NC). **(A)** qRT-PCR analysis of miR-25-3p expression levels in HT22 cells. **(B)** Cell viability was assessed at various time points using the CCK-8 assay. **(C)** Representative flow cytometry plots of intracellular Ca^2+^ levels detected by Fluo-4 a.m. staining. **(D)** Quantitative analysis of the mean fluorescence intensity of Ca^2+^. **(E)** Representative flow cytometry plots of the microglial activation marker CD16/32. **(F)** Quantification of the percentage of CD16/32-positive cells. Data are presented as mean ± SD (*n* = 3). **p < 0.01, ***p < 0.001, and ns indicates no significant difference.

To further confirm that the transfer of miR-25-3p from neurons to microglia is mediated by exosomes, we treated HT22 cells with GW4869, a well-known inhibitor of exosome release. The results demonstrated that GW4869 treatment significantly reduced the level of miR-25-3p in the exosomes derived from HT22 cells (Supplementary Figure S4A). Consequently, in the co-culture system, the elevation of miR-25-3p levels in BV2 cells induced by HT22 cells was remarkably abolished when HT22 cells were pre-treated with GW4869 (Supplementary Figure S4B). These data provide direct evidence that neuronal miR-25-3p is transferred to microglia via an exosome-dependent mechanism. Building upon this confirmed exosomal transfer mechanism, we next aimed to explore whether miR-25-3p in neuronal exosomes affects the function of microglia. To this end, we regulated the expression of miR-25-3p in HT22 cells by transfection in a Transwell co-culture system of HT22 and BV2 cells. To explore whether miR-25-3p in neuronal exosomes affects the function of microglia, we regulated the expression of miR-25-3p in HT22 cells by transfection in a Transwell co-culture system of HT22 and BV2 cells. The outcomes of qRT-PCR analysis revealed that, compared with the control group, the expression level of miR-25-3p in the miR-25-3p-OE group in HT22 cells was significantly increased, while it was significantly decreased in the miR-25-3p-KD group, successfully verifying the transfection efficiency of the miR-25-3p mimic and miR-25-3p inhibitor plasmids (Figure 2A). Functional experiments showed that knocking down miR-25-3p in neurons (miR-25-3p-KD) significantly inhibited the viability of BV2 cells (Figure 2B). In addition, it significantly enhanced the activation state of BV2 cells, as evidenced by an increase in both intracellular Ca^2+^ fluorescence intensity and the proportion of CD16/32-positive cells (Figures 2C–F).

**Figure 3:**
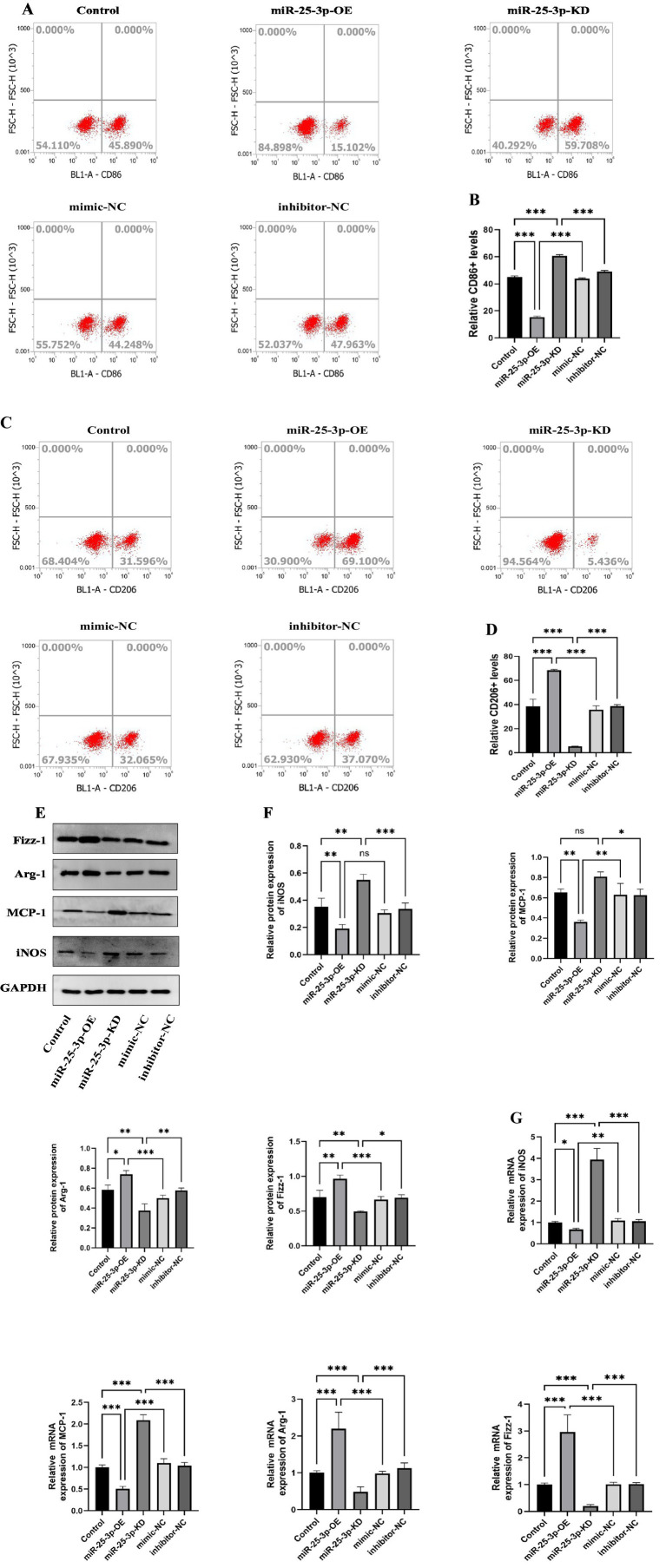
Neuronal exosomal miR-25-3p regulates M1/M2 polarization of BV2 microglia. **(A,B)** Flow cytometry analysis and quantification of the M1 marker CD86. **(C,D)** Flow cytometry analysis and quantification of the M2 marker CD206. **(E)** Western blot analysis of the protein expression of M1 markers (iNOS, MCP-1) and M2 markers (Arg-1, Fizz-1). **(F)** Quantitative analysis of relative protein expression levels. **(G)** qRT-PCR analysis of the relative mRNA expression levels of M1 and M2 markers. Data are presented as mean ± SD (*n* = 3). *p < 0.05, **p < 0.01, ***p < 0.001, and ns indicates no significant difference.

Given that the activation of microglia is closely related to their polarization direction, we further examined the markers related to M1/M2 polarization. Flow cytometry analysis revealed that the miR-25-3p-KD group markedly increased the expression of the M1 marker CD86, while concurrently reducing the levels of the M2 marker CD206. Conversely, the overexpression of miR-25-3p (miR-25-3p-OE) displayed a tendency to suppress M1 polarization and encourage M2 polarization (Figures 3A–D). Western blot and qRT-PCR confirmed this finding at the protein and mRNA levels, showing that miR-25-3p-KD led to a significant increase in the expression of M1 markers iNOS and MCP-1, and a reduction in the levels of M2 markers Arg-1 and Fizz-1 (Figures 3E–G). Taken together, these findings suggest that miR-25-3p derived from neuronal exosomes potently suppresses the polarization of microglia toward the pro-inflammatory M1 phenotype.

### 3.3 miR-25-3p inhibits the activation of the NF-κB signaling pathway by targeting TLR4

To clarify the molecular mechanism for how miR-25-3p regulates microglial polarization, we initially confirmed its potential targets using a dual-luciferase reporter gene assay. The data indicated that the miR-25-3p mimic caused a significant reduction in the luciferase activity of the reporter that carried the wild-type TLR4 3’UTR (TLR4-WT), but did not have a notable impact on the mutant type (TLR4-Mut), confirming that miR-25-3p is capable of directly binding to the 3’UTR of TLR4 (Supplementary Figure S1).

**Figure 4:**
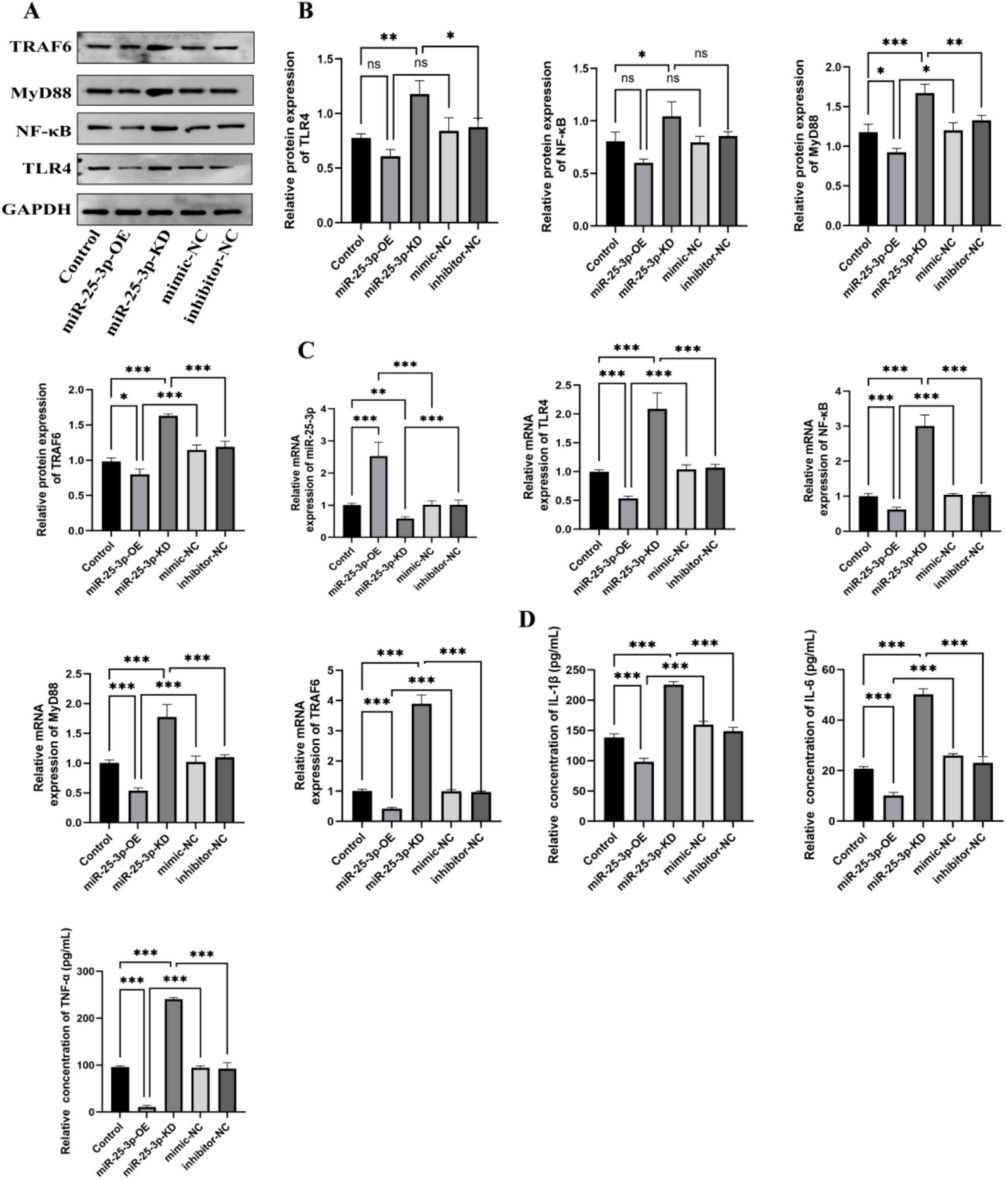
Neuronal exosomal miR-25-3p inhibits the TLR4/NF-κB signaling pathway and the production of pro-inflammatory cytokines in BV2 microglia. **(A)** Western blot analysis of the protein levels of TLR4, NF-κB, MyD88, and TRAF6. **(B)** Quantitative analysis of relative protein expression. **(C)** qRT-PCR analysis of the relative mRNA expression levels of pathway-related components. **(D)** ELISA detection of the concentrations of pro-inflammatory cytokines IL-1β, IL-6, and TNF-α in the cell culture supernatant. Data are presented as mean ± SD (*n* = 3). *p < 0.05, **p < 0.01, ***p < 0.001, and ns indicates no significant difference.

Based on this, we investigated the effect of miR-25-3p on the TLR4/NF-κB signaling pathway. In the co-culture system, after knocking down neuronal miR-25-3p, the protein expression levels and fluorescence intensities of TLR4 and its downstream signaling molecules—MyD88, TRAF6, and NF-κB—were all significantly increased in BV2 cells (Figures 4A–C; Supplementary Figures S2A–D). At the same time, the secretion levels of pro-inflammatory cytokines IL-1β, IL-6, and TNF-*α* were also significantly increased (Figure 4D). Conversely, overexpression of miR-25-3p markedly suppressed the activation of this pathway and curtailed the secretion of inflammatory mediators. These data provide strong evidence that miR-25-3p suppresses the inflammatory response of microglia through direct targeting of TLR4 and by preventing the activation of the MyD88/TRAF6/NF-κB signaling cascade that it orchestrates.

### 3.4 Neuronal exosomal miR-25-3p alleviates neurotoxicity by inhibiting microglial M1 polarization

**Figure 5:**
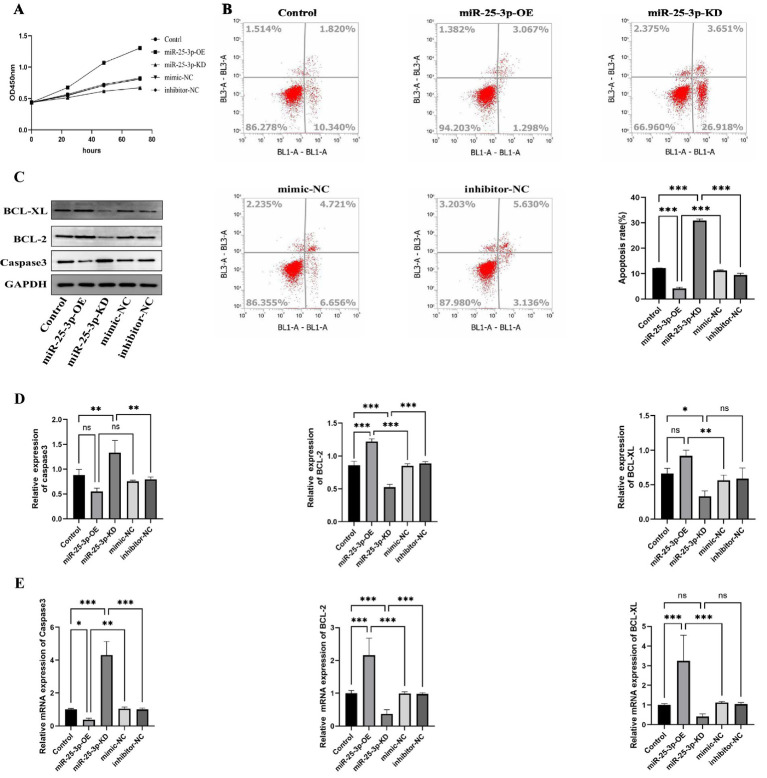
Neuronal exosomal miR-25-3p affects the proliferation and apoptosis of HT22 neurons by regulating microglia. **(A)** The viability of HT22 cells in different treatment groups was determined by the CCK-8 assay. **(B)** Apoptosis rate of HT22 cells detected by Annexin V-FITC/PI double staining flow cytometry and quantitative analysis. **(C)** Western blot detection of the expression of apoptosis-related proteins (Caspase-3, Bcl-2, Bcl-xL). **(D)** Quantitative analysis of the relative expression levels of apoptosis-related proteins. **(E)** qRT-PCR detection of the relative mRNA expression levels of apoptosis-related genes (Caspase-3, Bcl-2, Bcl-xL). Data are presented as mean ± SD (*n* = 3). *p < 0.05, **p < 0.01, ***p < 0.001, and ns indicates no significant difference.

To link the phenotypic changes of microglia with neuronal damage, we evaluated the survival status of HT22 neurons in the co-culture system. Data from CCK-8 and flow cytometry apoptosis analysis revealed that when miR-25-3p in neurons was knocked down, a significant reduction in the proliferative capacity of HT22 cells occurred, accompanied by a notable increase in the rate of apoptosis (Figures 5A,B). At the molecular level, there was an upregulation in the expression of the pro-apoptotic protein Caspase-3, whereas the levels of the anti-apoptotic proteins BCL-2 and BCL-XL were suppressed (Figures 5C–E).

**Figure 6:**
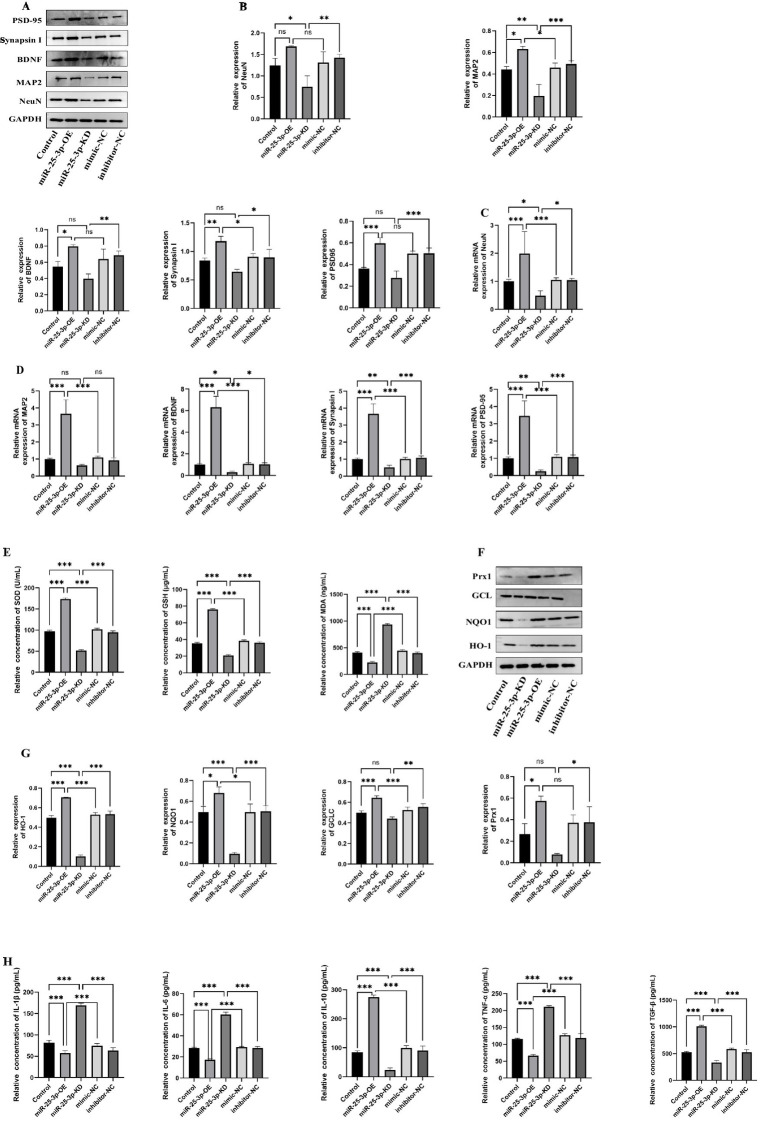
Effect of neuronal exosomal miR-25-3p on the function, oxidative stress, and inflammation of HT22 neurons. **(A)** Western blot detection of the expression of neuronal function markers (NeuN, MAP2, BDNF) and synapse-related proteins (Synapsin I, PSD-95). **(B)** Quantitative analysis of the relative expression levels of neural function-related proteins. **(C,D)** qRT-PCR detection of the relative mRNA expression levels of neural function-related genes. **(E)** ELISA detection of the concentrations of oxidative stress indicators (SOD, GSH, MDA). **(F)** Western blot detection of the protein expression of antioxidant factors (HO-1, NQO1, GCL, Prx1). **(G)** Quantitative analysis of the relative protein expression levels of antioxidant factors. **(H)** ELISA detection of the concentrations of inflammatory factors (IL-1β, IL-6, TNF-α, IL-10, TGF-β) in the HT22 cell culture supernatant. Data are presented as mean ± SD (*n* = 3). *p < 0.05, **p < 0.01, ***p < 0.001, and ns indicates no significant difference.

In addition, we also evaluated the function, oxidative stress, and inflammatory status of neurons. The findings revealed that within the miR-25-3p-KD group, the expression of neuronal function markers (NeuN, MAP2), neurotrophic factors (BDNF), and synapse-related proteins (Synapsin I, PSD-95) were all significantly downregulated (Figures 6A–D). Concurrently, there was an elevation in the level of intracellular oxidative stress, as evidenced by the decrease of antioxidants (SOD, GSH) and antioxidant factors (HO-1, NQO1, etc.), and the accumulation of lipid peroxidation products (MDA) (Figures 6E–G). Inflammatory factor detection showed that the concentrations of pro-inflammatory factors (IL-1*β*, IL-6, TNF-*α*) were elevated, while the concentrations of anti-inflammatory factors (IL-10, TGF-β) were diminished (Figure 6H). These findings suggest that a decrease in neuronal exosomal miR-25-3p induces significant neurotoxicity by promoting the M1 polarization of microglia, including inducing apoptosis, impairing neuronal function, and exacerbating oxidative stress and inflammatory responses.

### 3.5 TLR4 is a key molecule mediating microglial activation and M1 polarization

**Figure 7:**
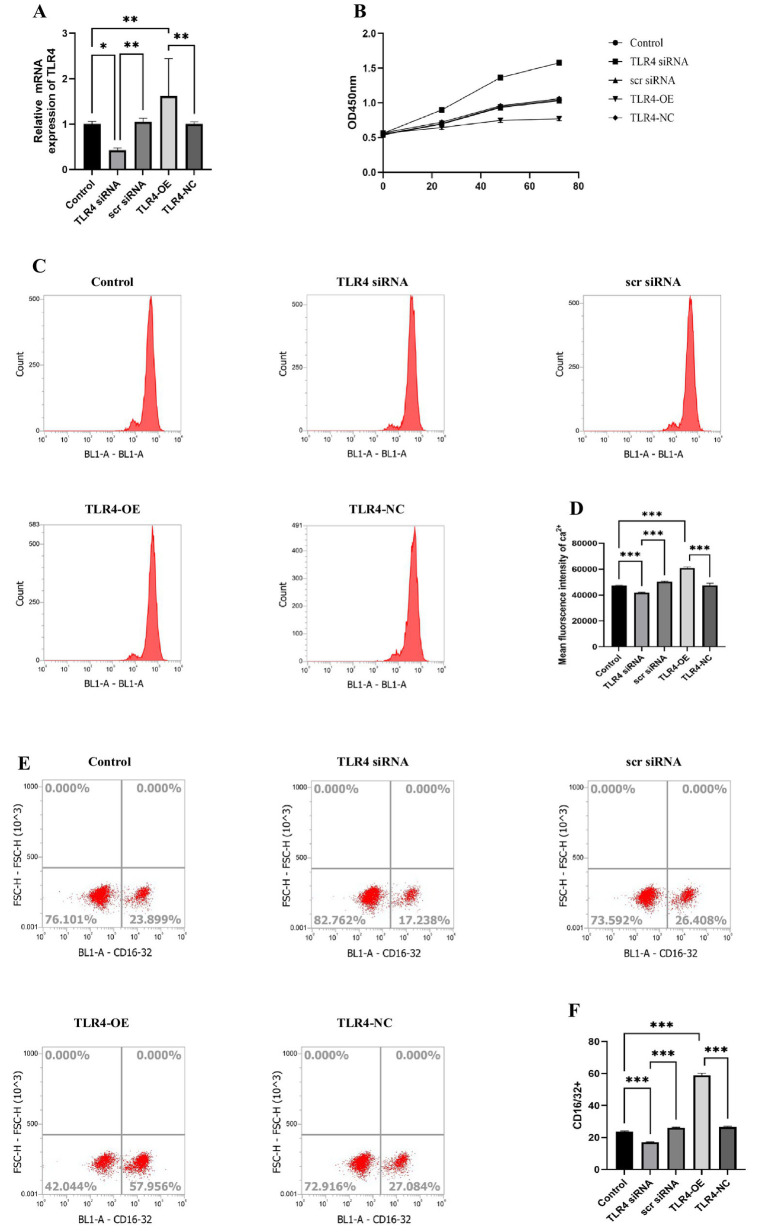
TLR4 mediates the activation of BV2 microglia. BV2 cells were transfected with TLR4 siRNA, scramble siRNA (scr siRNA), TLR4 overexpression plasmid (TLR4-OE), or an empty vector plasmid (TLR4-NC). **(A)** qRT-PCR analysis confirming TLR4 transfection efficiency. **(B)** Cell viability detected by CCK-8 assay. **(C)** Representative flow cytometry plots of intracellular Ca^2+^ levels detected by Fluo-4 a.m. staining. **(D)** Quantitative analysis of the mean fluorescence intensity of Ca^2+^. **(E)** Representative flow cytometry plots of the microglial activation marker CD16/32. **(F)** Quantification of the percentage of CD16/32-positive cells. Data are presented as mean ± SD (*n* = 3). *p < 0.05, **p < 0.01, ***p < 0.001, and ns indicates no significant difference.

To finally confirm the core role of TLR4 in the regulatory axis proposed in this study, we directly knocked down or overexpressed TLR4 in BV2 cells. qRT-PCR results successfully verified the transfection efficiency of TLR4 siRNA and the TLR4 overexpression plasmid (Figure 7A). Functional experiments showed that overexpression of TLR4 (TLR4-OE) significantly inhibited the viability of BV2 cells (Figure 7B). In addition, it significantly enhanced the activation of BV2 cells, as demonstrated by a rise in intracellular Ca2 + fluorescence intensity and an increased percentage of CD16/32 positive cells (Figures 7C–F), while knocking down TLR4 (TLR4 siRNA) had the opposite effect.

**Figure 8:**
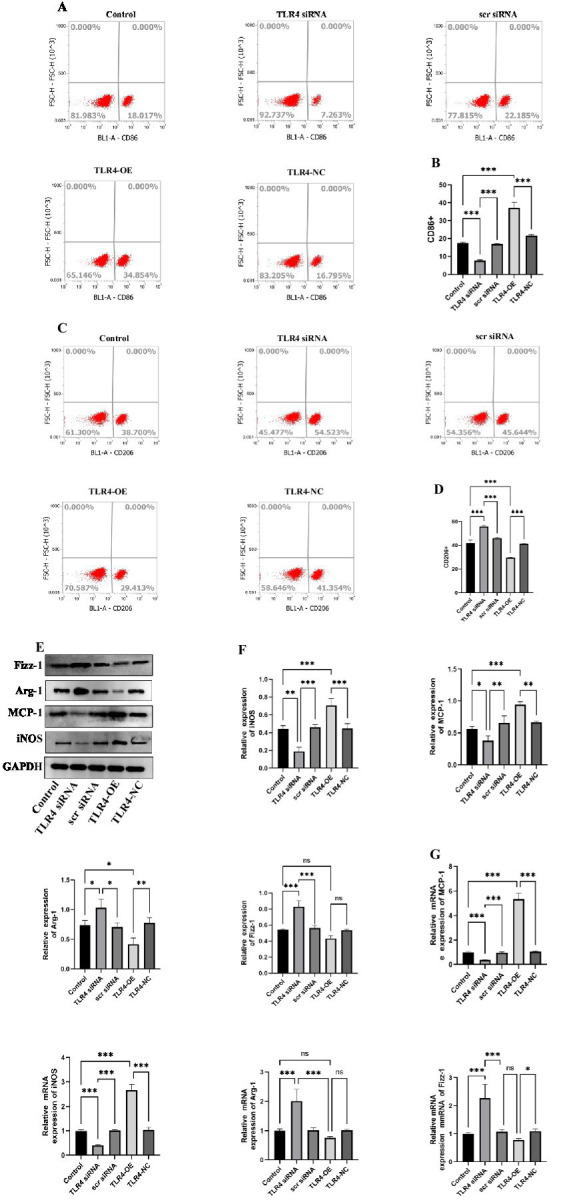
Regulation of M1/M2 polarization of BV2 microglia by TLR4. **(A,B)** Flow cytometry analysis and quantification of the M1 marker CD86. **(C,D)** Flow cytometry analysis and quantification of the M2 marker CD206. **(E)** Western blot analysis of the protein expression of M1 markers (iNOS, MCP-1) and M2 markers (Arg-1, Fizz-1). **(F)** Quantitative analysis of relative protein expression levels. **(G)** qRT-PCR analysis of the relative mRNA expression levels of M1 and M2 markers. Data are presented as mean ± SD (*n* = 3). *p < 0.05, **p < 0.01, ***p < 0.001, and ns indicates no significant difference.

In terms of polarization, flow cytometry analysis showed that TLR4-OE markedly elevated the expression of the M1 marker CD86, while concurrently reducing the levels of the M2 marker CD206 (Figures 8A–D). Western blot and qRT-PCR results further confirmed that TLR4-OE significantly increased the protein and mRNA levels of M1 markers iNOS and MCP-1, and decreased the expression of M2 markers Arg-1 and Fizz-1 (Figures 8E–G). These results collectively indicate that TLR4 is a key upstream molecule that drives microglial activation and polarization towards the M1 phenotype.

### 3.6 TLR4 promotes the release of pro-inflammatory cytokines by activating the NF-κB signaling pathway

**Figure 9:**
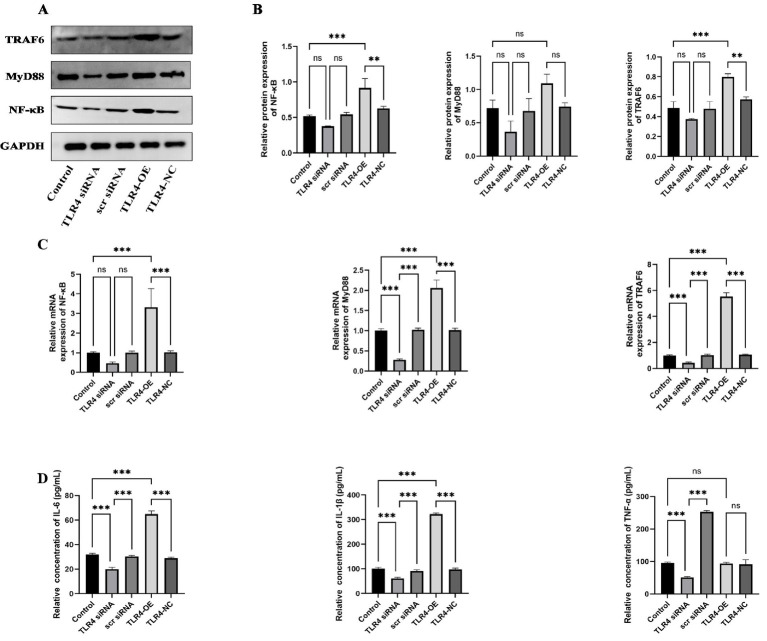
TLR4 regulates the NF-κB signaling pathway and the production of pro-inflammatory cytokines. **(A)** Western blot analysis of the protein levels of NF-κB, MyD88, and TRAF6. **(B)** Quantitative analysis of relative protein expression. **(C)** qRT-PCR analysis of the relative mRNA expression levels of pathway-related components. **(D)** ELISA detection of the concentrations of pro-inflammatory cytokines IL-6, IL-1β, and TNF-α in the cell culture supernatant. Data are presented as mean ± SD (*n* = 3). *p < 0.05, **p < 0.01, ***p < 0.001, and ns indicates no significant difference.

Next, we investigated the downstream mechanism by which TLR4 regulates microglial function. Immunofluorescence results intuitively showed that in the TLR4-OE group, the fluorescence intensity of the key proteins of the NF-κB pathway, MyD88, TRAF6, and NF-κB, were all significantly enhanced, indicating their upregulated expression (Supplementary Figure S3). Western blot and qRT-PCR quantitative analysis further confirmed that, in comparison to the control group, TLR4-OE markedly increased the expression of MyD88, TRAF6, and NF-κB at the protein and mRNA levels, while TLR4 siRNA significantly inhibited their expression (Figures 9A–C). As a functional consequence of the activation of this pathway, ELISA detection showed that BV2 cells in the TLR4-OE group released higher levels of the pro-inflammatory cytokines IL-6, IL-1β, and TNF-*α* (Figure 9D). These data indicate that TLR4 promotes the inflammatory response of microglia by activating the MyD88/TRAF6/NF-κB signaling pathway.

### 3.7 Inhibition of microglial TLR4 alleviates microglia-mediated neurotoxicity

**Figure 10:**
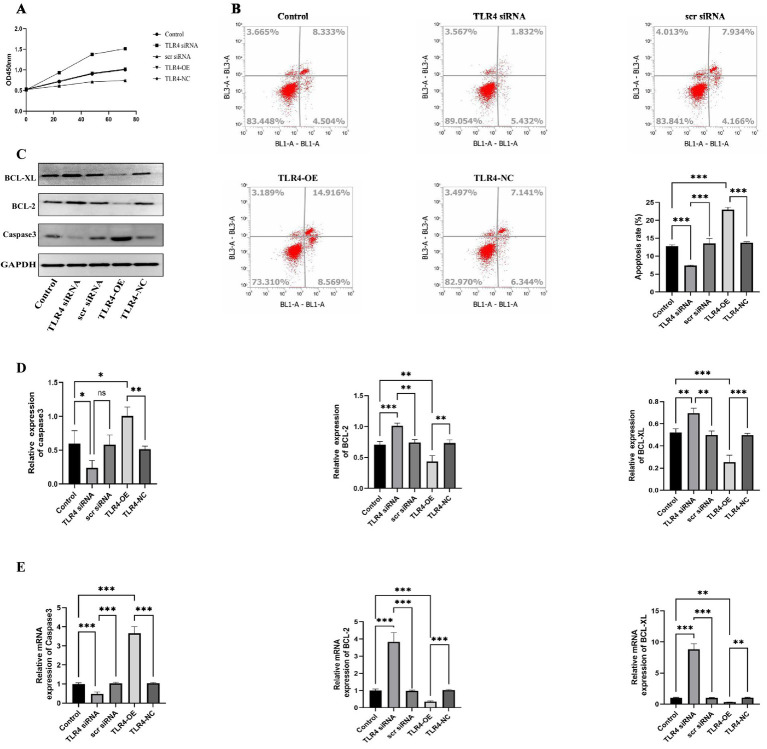
Modulation of TLR4 expression in microglia affects the proliferation and apoptosis of co-cultured HT22 neurons. **(A)** Proliferation viability of HT22 cells after co-culture in different treatment groups detected by CCK-8 assay. **(B)** Apoptosis rate of HT22 cells detected by Annexin V-FITC/PI double staining flow cytometry and quantitative analysis. **(C)** Western blot detection of the expression of apoptosis-related proteins (Caspase-3, Bcl-2, Bcl-xL). **(D)** Quantitative analysis of the relative expression levels of apoptosis-related proteins. **(E)** qRT-PCR detection of the relative mRNA expression levels of apoptosis-related genes (Caspase-3, Bcl-2, Bcl-xL). Data are presented as mean ± SD (*n* = 3). *p < 0.05, **p < 0.01, ***p < 0.001, and ns indicates no significant difference.

Finally, we directly linked the TLR4-mediated phenotypic changes of microglia with neuronal damage. In the HT22-BV2 co-culture system, when TLR4 was overexpressed in BV2 cells, HT22 neurons showed significant proliferation inhibition and increased apoptosis, with the apoptosis rate increasing from about 13% in the untreated group to about 23% (Figures 10A,B). At the molecular level, an upregulation in the expression of the pro-apoptotic protein Caspase-3 occurred, while the levels of the anti-apoptotic proteins BCL-2 and BCL-XL were suppressed (Figures 10C–E). Conversely, knocking down TLR4 in BV2 cells (TLR4 siRNA) could largely reverse the above neurotoxic effects and have a protective effect on neurons.

**Figure 11:**
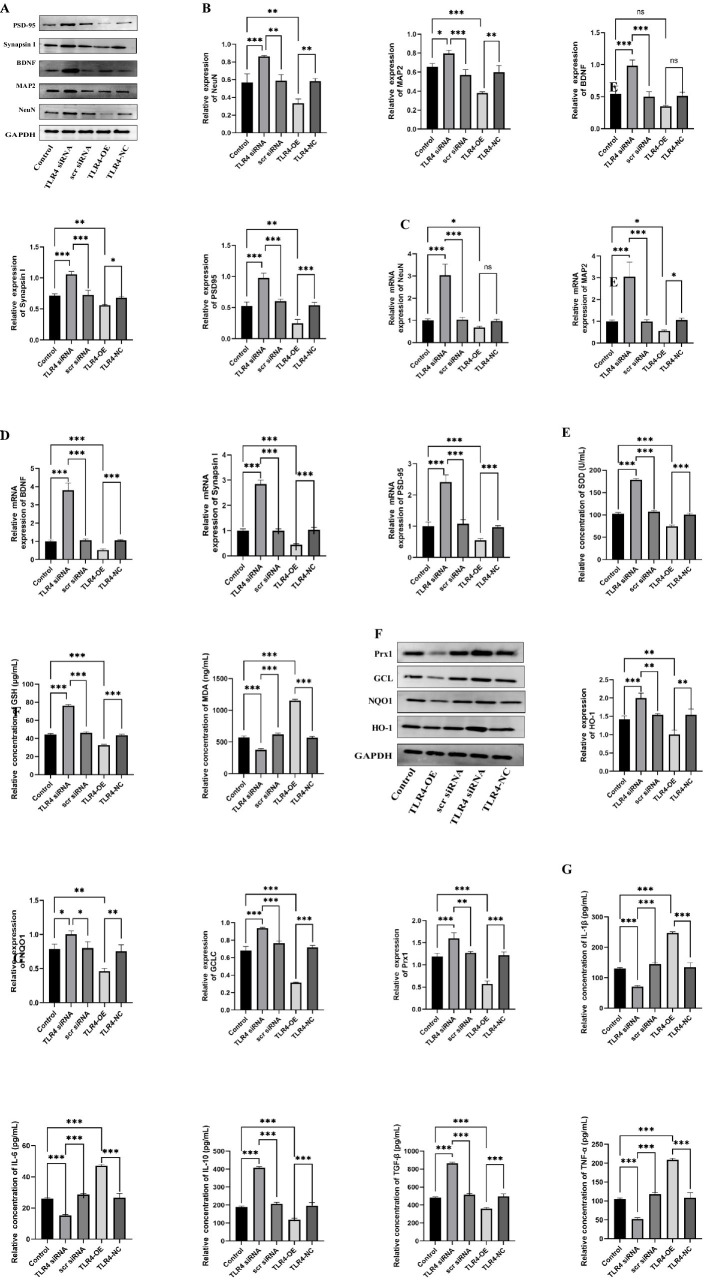
Inhibition of microglial TLR4 protects HT22 neurons against functional impairment, oxidative stress, and inflammation. **(A)** Western blot detection of the expression of neuronal function markers (NeuN, MAP2, BDNF) and synapse-related proteins (Synapsin I, PSD-95). **(B)** Quantitative analysis of the relative expression levels of neural function-related proteins. **(C,D)** qRT-PCR detection of the relative mRNA expression levels of neural function-related genes. **(E)** ELISA detection of the concentrations of oxidative stress indicators (SOD, GSH, MDA). **(F)** Western blot detection of the protein expression of antioxidant factors (HO-1, NQO1, GCL, Prx1). **(G)** ELISA detection of the concentrations of inflammatory factors (IL-1β, IL-6, TNF-α, IL-10, TGF-β) in the HT22 cell culture supernatant. Data are presented as mean ± SD (*n* = 3). *p < 0.05, **p < 0.01, ***p < 0.001, and ns indicates no significant difference.

Further functional evaluation showed that the function of HT22 neurons co-cultured in the TLR4-OE group was severely impaired, as evidenced by the comprehensive downregulation of the expression levels of neuronal markers (NeuN, MAP2), neurotrophic factors (BDNF), and synapse-related proteins (Synapsin I, PSD-95) (Figures 11A–D). At the same time, the neurons experienced severe oxidative stress, as evidenced by the depletion of antioxidants (SOD, GSH) and antioxidant factors (HO-1, NQO1, etc.), and the accumulation of lipid peroxidation products (MDA) (Figures 11E,F). In addition, the neurons in this group showed a stronger inflammatory state, with elevated concentrations of pro-inflammatory factors (IL-1β, IL-6, TNF-*α*) and diminished concentrations of anti-inflammatory factors (IL-10, TGF-β) (Figure 11G). These results finally established TLR4 as a key molecule that mediates M1 polarization of microglia and induces neurotoxicity, thereby validating the complete “neuronal exosomal miR-25-3p–TLR4/NF-κB–M1 microglial activation–neurotoxicity” signaling axis.

## 4 Discussion

Perioperative neurocognitive disorders (PND) have become a major challenge affecting the postoperative recovery and quality of life of elderly patients. Furthermore, neuroinflammation, which has microglial overactivation as its central element, has a crucial function in the onset and progression of this condition (18). This study systematically integrates and elucidates a novel neuron–microglia communication pathway, in which neurons deliver miR-25-3p via exosomes to suppress the activation of the TLR4/NF-κB signaling cascade in microglia, thereby preventing their polarization towards the pro-inflammatory M1 phenotype and ultimately achieving neuroprotection. This finding offers a novel viewpoint for comprehending the intricate pathological underpinnings of PND and emphasizes the importance of intercellular communication in maintaining central nervous system homeostasis.

Being the principal immune cells within the central nervous system, the dynamic balance of the functional state of microglia is crucial for the survival of neurons. In the pathological process of neurodegenerative diseases such as PND, the persistent M1 microglial activation is regarded as the primary element responsible for propelling neuroinflammation and neuronal damage (5, 6). Our research results are consistent with this view, indicating that inhibiting the M1 polarization of microglia can effectively alleviate neurotoxicity. In recent years, regulating the polarization state of microglia has become a hot strategy for PND intervention (19, 20). In this study, we found that neurons themselves can release protective signaling molecules (exosomal miR-25-3p) to suppress the M1 polarization of microglia, which suggests the existence of an endogenous neuroprotective mechanism. When this protective communication is disrupted (as in the knockdown of miR-25-3p in this study), the inflammatory response of these cells becomes uncontrolled, leading to neuronal injury. This presents a fresh rationale for the development of PND: that is, PND may not only be due to the enhancement of inflammatory attacks, but also due to the weakening of endogenous anti-inflammatory and repair signals.

The TLR4/NF-κB signaling cascade is a well-established pathway that mediates innate immunity and inflammatory responses, and plays a pivotal role in the M1 polarization of microglia (11, 21). A large number of studies have confirmed that this pathway is significantly activated in models of anesthesia, surgical trauma, or neurodegenerative diseases, and is closely related to cognitive dysfunction (22, 23). Through a dual-luciferase reporter gene assay, this study further verified that miR-25-3p directly targets and inhibits the expression of TLR4, thus providing a new upstream mechanism for the regulation of this pathway. Our results show that the reduction of neuron-derived miR-25-3p leads to the upregulation of TLR4 and its downstream molecules MyD88 and TRAF6 in microglia, and ultimately promotes the nuclear translocation of NF-κB p65 and the transcription of pro-inflammatory genes. This finding not only enriches the understanding of the regulatory network of the TLR4/NF-κB pathway, but also highlights the potential of miR-25-3p as an endogenous TLR4 inhibitor. Consistent with recent research findings that targeting the TLR4 pathway can effectively inhibit microglial activation and provide neuroprotection (24, 25), our work further clarifies that miR-25-3p is one of the key molecules to achieve this regulation. A previous study by Luo et al. also indicated that miR-25-3p could target the TLR4 axis in sepsis-associated encephalopathy (16). However, our study uniquely highlights the exosome-mediated intercellular transfer of miR-25-3p from neurons to microglia as a critical regulatory mechanism in the context of neuroinflammation.

Extracellular vesicles, especially exosomes, as carriers of intercellular information transmission, have received much attention in the field of neuroscience in recent years (14). They can cross the blood–brain barrier, deliver their loaded bioactive molecules such as miRNAs and proteins, and regulate the function of target cells (26). The exosome-mediated communication between neurons and microglia is considered to be an important way to regulate neuroinflammation and synaptic plasticity (27, 28). For example, it has been reported that neuron-derived exosomes can carry miR-21-5p or miR-9-5p to promote the M1 polarization of microglia (29, 30). Our study, however, identifies an opposing, neuroprotective pathway wherein neurons release miR-25-3p via exosomes to suppress M1 polarization. This indicates that neuron–microglia communication is bidirectional and complex, and its final effect may depend on the specific miRNA species loaded in the exosomes and the pathological microenvironment. Recent studies have also confirmed that exosomes derived from M2 microglia can exert neuroprotective effects through miR-25-3p (31), which forms an interesting complement to our findings, suggesting that miR-25-3p may be a universal anti-inflammatory and repair signaling molecule in the central nervous system.

The findings of this study have important clinical translational potential. First, the level of miR-25-3p in neuronal exosomes may become a potential biomarker for assessing the risk of PND or the severity of the disease. Second, supplementing with miR-25-3p mimics or delivering engineered exosomes rich in miR-25-3p through intravenous injection may potentially serve as a novel cell-free therapeutic strategy for PND, although this warrants further *in vivo* investigation. This strategy aims to restore endogenous neuroprotective communication and control neuroinflammation by targeting the polarization state of microglia. Compared with traditional broad-spectrum anti-inflammatory drugs, it may have higher specificity and fewer side effects (32, 33).

However, this study also has some limitations. First, this study is mainly based on an *in vitro* cell co-culture model. Although it can clearly reveal the molecular mechanism, it cannot fully simulate the complex pathophysiological environment in vivo. Future research should further verify the in vivo function and therapeutic effect of the “neuronal exosomal miR-25-3p/TLR4” signaling axis in PND animal models (such as elderly mice undergoing laparotomy or sevoflurane anesthesia). To bridge this gap, our subsequent studies will utilize stereotaxic injection of engineered exosomes or specific miRNA inhibitors in animal models to validate these findings in a more physiologically relevant context. Second, this study only focused on miR-25-3p, a single miRNA, while exosomes may contain a variety of synergistic molecules, and their overall effect needs to be systematically analyzed by high-throughput sequencing and other technologies. In addition, the upstream regulatory mechanism of how neurons increase or decrease the secretion of miR-25-3p exosomes under what stimuli is still unclear and deserves in-depth investigation. Furthermore, the use of immortalized cell lines (HT22 and BV2) represents a notable limitation. While these cell lines are widely employed in neurobiological research, BV2 cells may not fully recapitulate the transcriptional states of primary microglia (34), and HT22 cells lack certain features of mature neurons. To address this, we aim to validate our key findings using primary cultured murine microglia and neurons, or human induced pluripotent stem cell (iPSC)-derived neural cells, which better reflect primary cell biology. Additionally, the physiological ratio of microglia to neurons in the central nervous system is complex and region-dependent (35), which may not be perfectly modeled by our fixed *in vitro* cell density (HT22 cells at 1 × 10^5^ cells/well and BV2 cells at 5 × 10^4^ cells/well). Future in vitro studies will incorporate varying cell ratios or utilize 3D brain organoid models to more accurately simulate the intricate spatial and numerical relationships between neurons and microglia in the CNS.

In summary, this study systematically elucidates the molecular mechanism by which neurons target and inhibit the microglial TLR4/NF-κB signaling pathway through exosomal miR-25-3p, thereby inhibiting M1 polarization and alleviating neurotoxicity. This finding not only deepens our understanding of the role of neuron–glia communication in PND, but also provides a new theoretical basis and promising therapeutic targets for the development of precise intervention strategies for PND.

## Data Availability

The original contributions presented in the study are included in the article/Supplementary material, further inquiries can be directed to the corresponding author.
