# Supplementary material for: Neuronal exosomal miR-25-3p attenuates M1 microglial activation and neurotoxicity by targeting TLR4 to regulate the NF-κB signaling pathway
Source: Front Neurol. 2026 Jun 3;17:1803653. doi: 10.3389/fneur.2026.1803653 (PMC13277419; doi:10.3389/fneur.2026.1803653)
Supplement: Supplementary file 1 [file Supplementary_file_1.DOC]

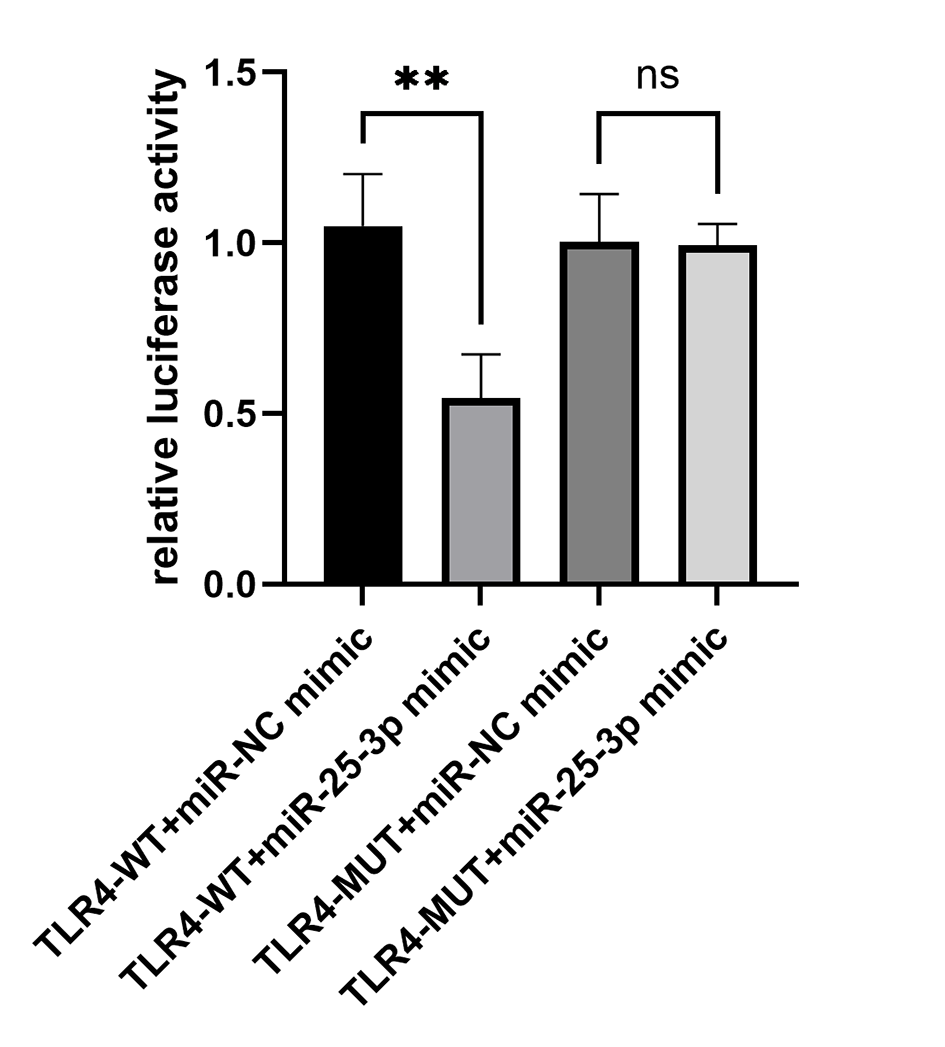


**Figure S1.** Dual-luciferase reporter assay verifying the direct targeting relationship between miR-25-3p and the TLR4 3'UTR. Data are presented as mean ± SD (n=3). *P < 0.05, **P < 0.01, ns indicates no significant difference.


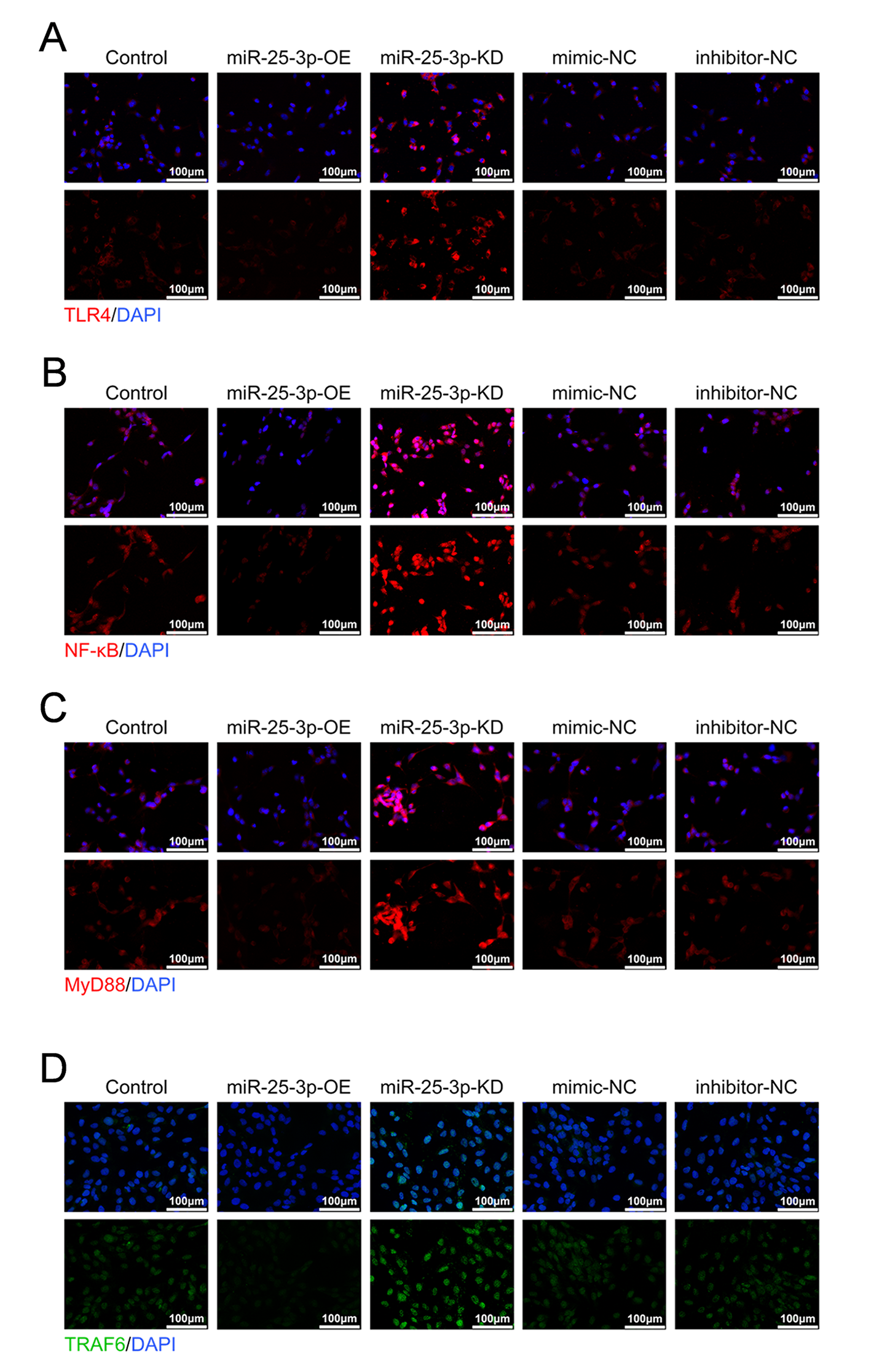


**Figure S2. Immunofluorescence analysis of the TLR4/NF-κB pathway in BV2 microglia following miR-25-3p modulation.** Representative immunofluorescence images showing the expression and localization of (A) TLR4 (red), (B) NF-κB (red), (C) MyD88 (red), and (D) TRAF6 (green) in different treatment groups. Nuclei were counterstained with DAPI (blue). Scale bar = 100 μm.


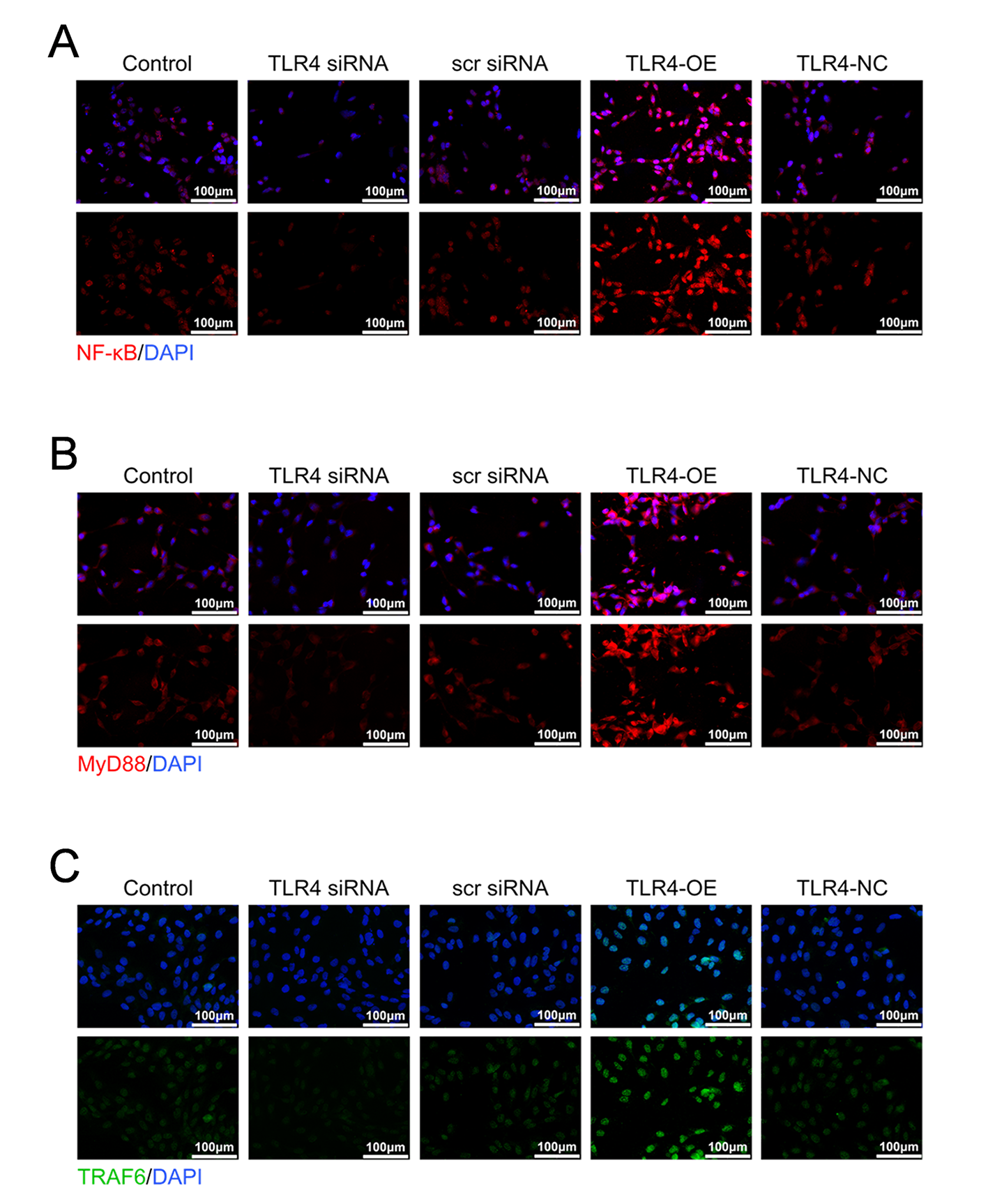


**Figure S3. Immunofluorescence analysis of NF-κB pathway-associated proteins following TLR4 modulation.** Representative immunofluorescence images showing the expression and localization of (A) NF-κB (red), (B) MyD88 (red), and (C) TRAF6 (green) in different treatment groups. Nuclei were counterstained with DAPI (blue). Scale bar = 100 μm.


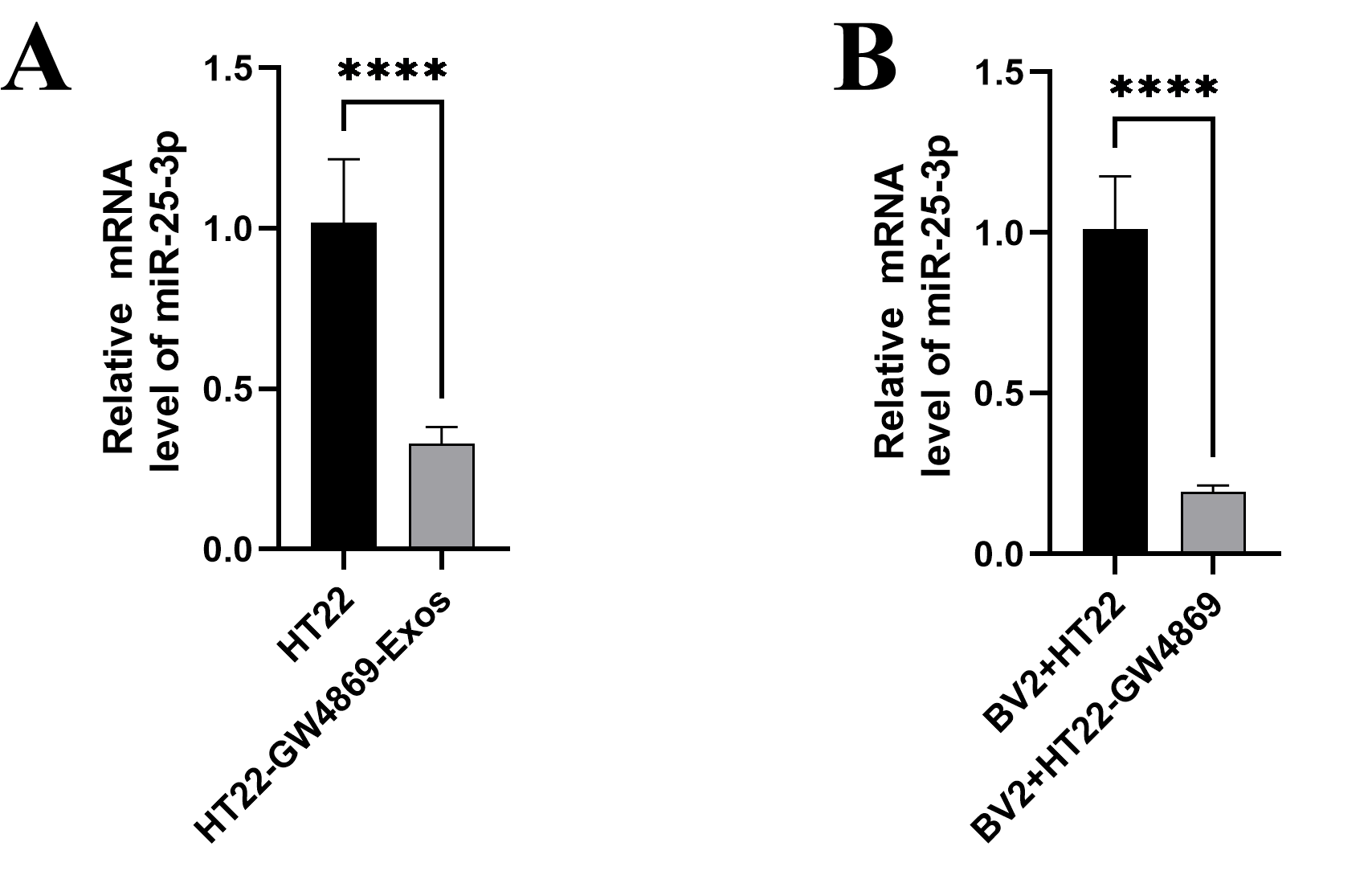


**Figure S4. Verification of exosome-mediated transfer of miR-25-3p using GW4869.**(A) qRT-PCR analysis of miR-25-3p levels in exosomes extracted from HT22 cells treated with or without the exosome inhibitor GW4869 (20 μM). (B) qRT-PCR analysis of miR-25-3p levels in BV2 cells co-cultured with HT22 cells, with or without GW4869 pre-treatment. Data are presented as mean ± SD (n=3). ****P < 0.0001**.**
